# AHR signaling pathway mediates mitochondrial oxidative phosphorylation which leads to cytarabine resistance: AHR pathway mediates mitochondrial oxidative phosphorylation

**DOI:** 10.3724/abbs.2024022

**Published:** 2024-02-26

**Authors:** Yan Jia, Xiyu LI, Lulu Chen, Ling Li, Suzhen zhang, Wenhui Huang, Hao Zhang

**Affiliations:** 1 Department of Hematology Affiliated Hospital of Jining Medical University Jining 272000 China; 2 Shangdong University of Traditional Chinese Medicine Jinan 250011 China; 3 Department of Clinical Medicine Jining Medical University Jining 272000 China

**Keywords:** AHR expression, AML, mitochondrial oxidative phosphorylation, cytarabine resistance

## Abstract

The aryl hydrocarbon receptor (AHR) has been identified as a significant driver of tumorigenesis. However, its clinical significance in acute myeloid leukemia (AML) remains largely unclear. In this study, RNA-Seq data from AML patients (bone marrow samples from 173 newly diagnosed AML patients) obtained from the TCGA database, and normal human RNA-Seq data (bone marrow samples from 70 healthy individuals) obtained from the GTEX database are downloaded for external validation and complementarity. The data analysis reveals that the AHR signaling pathway is activated in AML patients. Furthermore, there is a correlation between the expressions of AHR and mitochondrial oxidative phosphorylation genes. *In vitro* experiments show that enhancing AHR expression in AML cells increases mitochondrial oxidative phosphorylation and induces resistance to cytarabine. Conversely, reducing AHR expression in AML cells decreases cytarabine resistance. These findings deepen our understanding of the AHR signaling pathway’s involvement in AML.

## Introduction

The aryl hydrocarbon receptor (AHR), a cytosolic ligand-activated transcription factor, is expressed widely in various vertebrate cell types [ 1, 2]. Numerous studies have demonstrated the involvement of AHR in multiple signaling pathways that regulate diverse physiological processes, including cell proliferation, differentiation, motility, gene regulation, and migration [3]. There is increasing evidence suggesting that AHR activation plays critical roles in tumorigenesis, including metastasis, initiation, promotion, and progression [ 4‒ 6]. Several members of the kynurenine pathway, a key process in tryptophan catabolism, are recognized as endogenous ligands for AHR [ 7‒ 9]. The conversion of Trp to N-formyl-L-kynurenine, a crucial step in the kynurenine pathway, is mediated by two rate-limiting enzymes: tryptophan 2,3-dioxygenases (TDO) and indoleamine 2,3-dioxygenases (IDO) [7]. A study by Ahmed *et al*. [10] revealed that interleukin-4-induced-1 (IL4I1) was more frequently associated with AHR activity than IDO1 or TDO2, which were previously recognized as the main Trp-catabolic enzymes. Endogenous AHR ligands are elevated in cancer patients and have been correlated with survival outcomes [ 11‒ 13]. Activation of AHR regulates the expressions of cytochromes P450 (CYP1A1, 1A2, and 1B1), and increased expression levels of these cytochromes represent AHR activation [ 7, 13, 14].

The Warburg effect, which demonstrates that cancer cells exhibit higher rates of glycolysis, leads to the belief that mitochondria are completely dysfunctional in cancer, with low levels of oxidative phosphorylation (OXPHOS) [ 15‒ 17]. However, recent studies have shown that while cancer cells undergo increased glycolysis, their mitochondrial oxidative phosphorylation plays crucial roles in meeting the bioenergetic and macromolecular anabolic needs of cancer cells. This helps maintain cancer cell stemness, promotes proliferation, and facilitates metastasis [ 18, 19]. Numerous studies have now demonstrated the requirement of OXPHOS for tumor cell immortalization [20]. Mitochondrial oxidative phosphorylation also plays significant roles in cancer cell growth and drug resistance. Inhibiting mitochondrial oxidative phosphorylation has been shown to promote apoptosis and reduce drug resistance in tumor cells [21]. There is growing interest in targeting OXPHOS and the electron transport chain for cancer therapy [ 22‒ 24]. Previous studies have found that chemotherapy-resistant human acute myeloid leukemia (AML) cells exhibit increased OXPHOS activity [25]. Subsequently, Yucel *et al*. [26] confirmed that suppressing oxidative phosphorylation sensitizes leukemia cell lines to cytarabine, a chemotherapy drug.

There have been numerous studies highlighting the association between the AHR and OXPHOS [ 27, 28]. Activation of AHR has been found to be linked to the enzymatic activities of ATP synthase and the electron transport chain (ETC) in the OXPHOS system [29]. Additionally, AHR has been shown to regulate mitochondrial function and protect the myocardium against myocardial ischemia-reperfusion injury [30]. Previous studies revealed that AHR signaling plays a crucial role in modulating mitochondrial activity and oxidative stress, which are important for maintaining cellular homeostasis [ 31, 32].

In the present study, we conducted bioinformatics analysis and *in vitro* experiments to investigate the role of the AHR in acute myeloid leukemia (AML). Our findings suggested that AHR expression is correlated with mitochondrial oxidative phosphorylation, which could potentially contribute to cytarabine resistance. These insights provide a foundation for further exploring the molecular mechanisms involved and identifying potential therapeutic targets related to AHR in AML.

## Materials and Methods

### RNA-sequencing data and bioinformatics analysis

AML RNA-Seq (bone marrow of 173 newly diagnosed AML patients) from the TCGA database and normal human RNA-Seq from the GTEX database (bone marrow of 70 normal people) were downloaded at https://xenabrowser.net/datapages and transformed via log(x+1). The clinical information of TCGA-LAML patients was downloaded from the website ( https://www.cancer.gov/about-nci/orga nization/ccg/research/structural-genomics/tcga). This study was in full compliance with the published guidelines of TCGA and GTEx.

### Differentially expressed gene (DEG) analysis

R package LIMMA (linear models for microarray data) was adopted to analyze the difference of gene expressions. Each gene expression was calculated based on the false discovery rate (FDR; *P*<0.05) using the Benjamini-Hochberg method and *t*-test. The DESeq2 R package was constructed to compare expression data of low- and high-expression of AHR signaling pathway genes (cut-off value of 50%) in AML samples (HTseq-Count) to identify DEGs.

### Survival analysis

To individualize the correlations between the expressions of DEGs and the overall survival of AML patients, a nomogram was generated by using the RMS R package (version 5.1-3), which included prominent clinical characteristics and calibration plots. The statistical significance of the correlation was tested by the log-rank test. In addition, the C-index and receiver operating characteristic (ROC) curve were used to analyze and compare the predictive accuracy of the nomogram and separate prognostic factors.

### Patients

Fresh human bone marrow and peripheral blood were obtained from the Department of Hematology in the Affiliated Hospital of Jining Medical University. The patients with AML were diagnosed according to the French-American-British classification system. The present study was approved by the Affiliated Hospital of Jining Medical University. All participants signed informed consent forms prior to inclusion in the study.

### Cell lines and cell culture

The Skm1 cell line (from Dr Xiang Li, Shanghai Jiao Tong University, Shanghai, China) and SHI-1 and U937 cell lines (Tongpai Company, Shanghai, China) were used in this study. Skm1, SHI-1, and U937 cells were cultured in RPMI-1640 medium (Jiruo Company, Hangzhou, China) supplemented with 10% fetal bovine serum and 5% penicillin-streptomycin at 37°C in a humidified atmosphere with 5% CO _2_. The cells were seeded at a density of 2×10 ^5^ cells/mL, and different concentrations of SR1 (Selleck, Shanghai, China) were used to inhibit AHR activation. L-Kyn (Santa Cruz Biotech, Santa Cruz, USA) was used to activate AHR. Cytarabine (Selleck) was used in the study.

### Real-time quantitative polymerase chain reaction (RT-qPCR)

Total RNA was extracted using the RNeasy Mini kit (Qiagen, Hilden, Germany), and cDNA was synthesized utilizing the PrimeScript RT reagent kit (TaKaRa, Dalian, China). Both procedures were carried out according to the manufacturers’ protocols. Total RNA (<1 μg) from each sample was used to synthesize complementary (c)DNA . The RT reaction was performed at 37°C for 15 min, followed by 85°C for 5 s. Quantitative polymerase chain reaction (qPCR) was conducted using Real-time PCR Master Mix (TaKaRa) on an ABI7500 real-time PCR system (Applied Biosystems, Foster City, USA). For amplification, an initial denaturation step at 95°C for 30 s was followed by 40 cycles at 95°C for 5 s, 60°C for 15s, and 60 °C for 34 s. The sequences of primers used in this study are shown in Table 1.

**Table:** **Table 1** The sequences of primers used in this study

Gene	Sequence (5′→3′)
*β-actin*	F: CCTTCCTGGGCATGGAGTCCTG
	R: GGAGCAATGATCTTGATCTTC
*AHR*	F: ACATCACCTACGCCAGTCGC
	R: TCTATGCCGCTTGGAAGGAT
*CYP1A1*	F: GTCTTGGACCTCTTTGGAGCT
	R: GTGACCTGCCAATCACTGTG
*CYP1B1*	F: CACCTCTGTCTTGGGCTACC
	R: TTCGCAGGCTCATTTGGGTT
*FH*	F: TGGGAATCCAGGCCAATAC
	R: GCTGCCTTGTCATACCCTAT
*ACO2*	F: GAGCTGAAGCCACACATCAA
	R: GAGCGCCCCATATCTTCATA
*CS*	F: ACCTGTCAGCGAGAGTTTGC
	R: CCCAAACAGGACCGTGTAGT
*OGDH*	F: GAGGCTGTCATGTACGTGTGCA
	R: TACATGAGCGGCTGCGTGAACA
*PDHB*	F:AAGAGGCGCTTTCACTGGAC
	R: ACTAACCTTGTATGCCCCATCA
*PDHX*	F:TTGGGAGGTTCCGACCTGT
	R: CAACCACTCGACTGTCACTTG

### Western blot analysis

Cells were lysed on ice for 10‒20 min in RIPA buffer (Gibco, Carlsbad, USA). Cell lysates were then centrifuged (12,000 *g*), and supernatants were collected for western blot analysis. Equal amounts were separated by SDS-PAGE and blotted onto PVDF membranes (Millipore, Billerica, USA). The membranes were initially incubated with primary antibodies including anti-AHR antibody (Abcam, Cambridge, UK), anti-CYP1A1 antibody (Abcam), and anti-CYP1B1 antibody (Abcam), subsequently incubated with the corresponding secondary antibodies for 1 h. Specific bands were visualized using an Enhanced chemiluminescence (ECL) western blotting detection kit (Amersham Biosciences, Buckinghamshire, UK).

### Complex enzymatic activities

ETC ComplexI enzyme activity (ab109721; Abcam) and ETC Complex II enzyme activity (ab109908; Abcam) were measured using kits according to the manufacturer’s protocol. Samples were normalized according to the protein amount measured by BSA assay. Multiskan Sky microplate reader (Thermo Fisher Scientific, Waltham, USA) was used to detect colorimetric enzyme activity.

### Viral transfection assay

shAHR and AHR-OE lentiviruses were purchased from Genechem Company (Shanghai, China). Transfection was performed according to the manufacturer’s protocol. The helper transfection reagent and the appropriate amount of virus were added to 2×10 ^5^/mL cells. It was cultured in an incubator at 37°C with 5% CO _2_ for 8 h, and then fresh medium was added. After culture for 3‒4 days, western blot analysis and PCR were used to evaluate the transfection efficiency.

### Apoptosis assay

The apoptosis assay was performed using a 7-AADD/annexin Kit (Multi Sciences Biotech Co., Ltd, Hangzhou, China). Flow cytometry was used to detect the apoptosis rate according to the manufacturer’s instructions. Annexin V/PE and 7-AADD were added into 100 μL cell suspension then incubated for 15 min at room temperature (25°C) in the dark. The apoptotic cells were examined using a flow cytometer (Becton, Dickinson, Franklin Lakes, USA).

### Viability assay

Briefly, a mixture of 0.4% trypan blue (Solarbio, Beijing, China) and a one-part cell suspension (1:9) was prepared. After 3 min, a drop of the mixture was applied to a hemocytometer. The average number of total cells and unstained (viable) cells was determined. The rate of cell viability was determined as follows: average viable cell count/average total cell count×100%.

### Statistical analysis

R language (v3.6.2) was used for statistical analysis. *P* values of the difference in gene expression between normal tissues and AML were calculated using the Wilcoxon signed rank test. *P* values of Kaplan-Meier survival analysis were calculated using the log-rank test. Statistical analysis was performed with SPSS 13.0 software (SPSS, Inc., Chicago, USA). The data were analyzed with Student’s *t* test or one-way analysis of variance. A *P* value less than 0.05 was considered statistically significant.

## Results

### Molecules related to the AHR signaling pathway are increased in patients with AML

**Figure 1:**
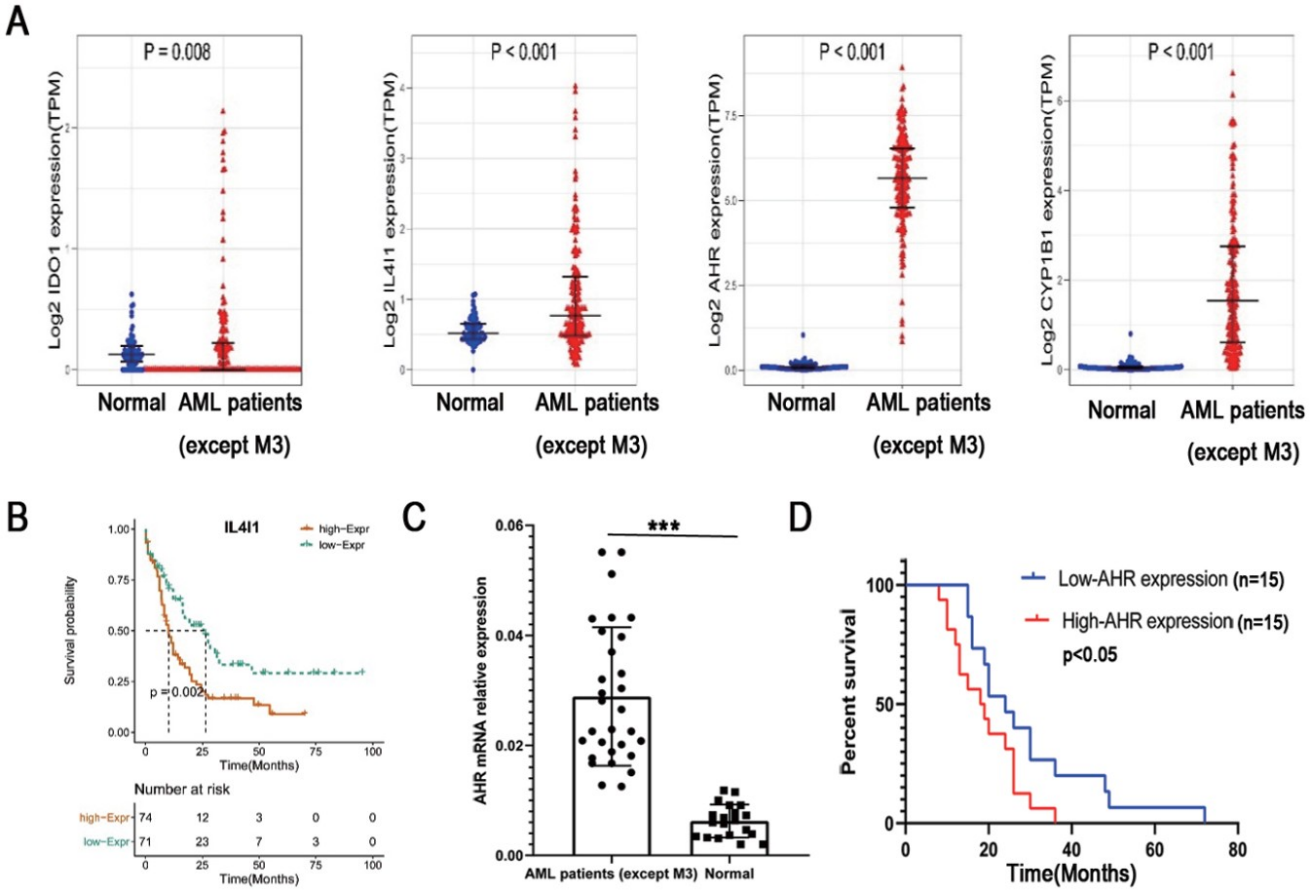
Figure 1 AHR signaling pathway molecules are highly expressed in patients with acute myeloid leukemia (AML) (A) The expressions of AHR signaling pathway genes (IL-4i1, AHR, CYP1A1, and CYP1B1) were determined in AML patients compared with those in normal individuals. To assess the prognostic significance of AHR signaling gene expression levels, we investigated the overall survival of TCGA-LAML patients. (B) High expression of the AHR signaling gene IL4-i1 was correlated with poor overall survival. (C) To further investigate the role of AHR in AML patients, we assessed AHR expression in bone marrow and its relationship with AML survival. The results indicated higher AHR expression in AML patients relative to normal individuals. (D) The elevated expression of AHR in AML patients was associated with worse survival outcomes. ***P<0.001.

The Kynurenine pathway plays a crucial role in activating AHR, which is mediated by IDO1 and IL4i1. Increased expressions of CYP1A1 and CYP1B1 are widely considered a major marker of AHR activation. To detect the AHR signaling pathway, we analyzed their oncoprints in AML (TCGA, Pan-AML) using cBioPortal. As shown in Figure 1A, the expressions of AHR signaling pathway genes ( *IL-4i1*, *AHR*, *CYP1A1*, and *CYP1B1*) are significantly higher in AML patients compared to those in normal individuals. To evaluate the prognostic value of AHR signaling gene expression levels, we examined the overall survival of TCGA-LAML patients. The clinical information of TCGA-LAML patients was downloaded from the website https://www.cancer.gov/about-nci/orga nization/ccg/research/structural-genomics/tcga. The DESeq2 R package was utilized to compare expression data of low- and high-expression of AHR signaling pathway genes (cut-off value of 50%) in AML samples (HTseq-Count) to identify differentially expressed genes (DEGs). The results showed that IL4i1 expression was associated with poor overall survival ( Figure 1B). To further elucidate the role of AHR in AML, we examined the expression of AHR in bone marrow and explored the relationship between AHR and AML survival. The expression level of AHR in bone marrow mononuclear cells (BMMCs) was significantly higher in AML patients than in normal individuals and was found to be linked to a worse overall survival rate ( Figure 1C,D). These findings suggested that AHR may have implications for AML progression and patient outcomes.

### The expression of AHR shows a positive correlation with the genes encoding the rate-limiting enzymes of the mitochondrial tricarboxylic acid cycle

**Figure 2:**
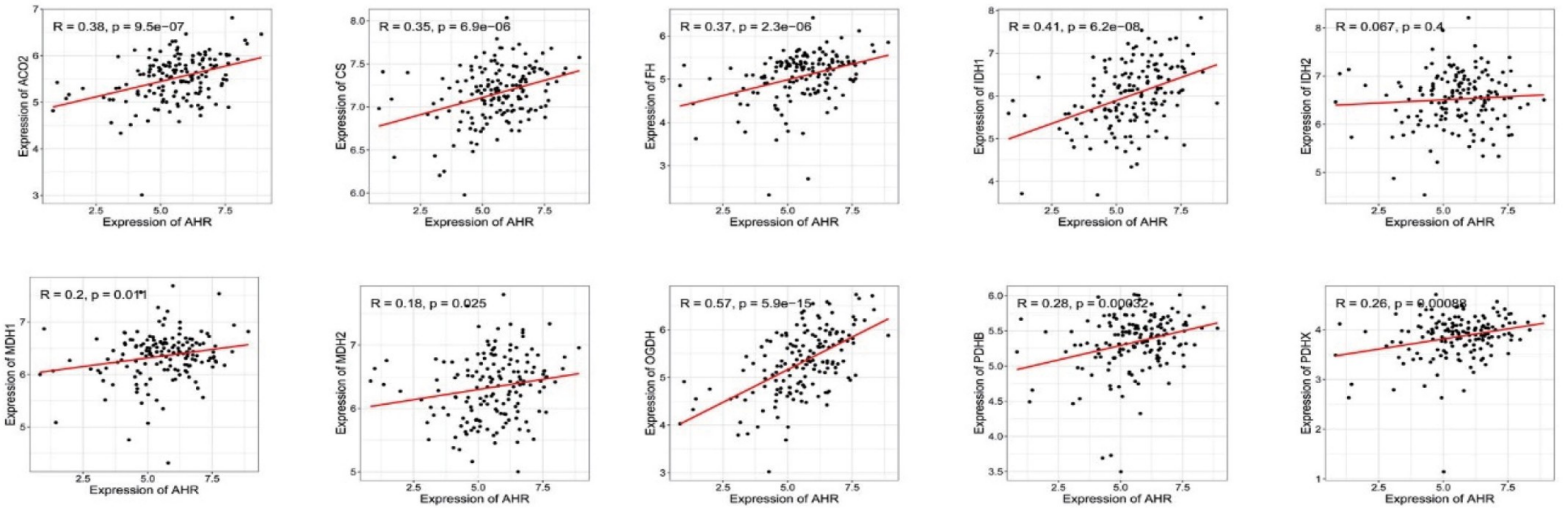
Figure 2 The expression of AHR is positively associated with the genes encoding the key enzymes of the mitochondrial tricarboxylic acid (TCA) cycle The clinical information of TCGA-LAML patients was downloaded from the website (https://www.cancer.gov/about-nci/orga nization/ccg/research/structural-genomics/tcga). According to the data analysis, we found that AHR expression was positively associated with the rate-limiting enzyme genes of the mitochondrial tricarboxylic acid cycle.

Based on the bioinformatics analysis, it was found that AHR expression showed a positive association with the genes encoding key enzymes of the mitochondrial tricarboxylic acid cycle. The correlation coefficients (R values) between AHR expression and the respective genes were as follows: ACO2 (R=0.38), CS (R=0.35), FH (R=0.37), IDH1 (R=0.41), MDH1 (R=0.2), MDH2 (R=0.18), OGDH (R=0.57), PDHB (R=0.28), and PDHX (R=0.26) ( Figure 2 **)**. This suggests a potential relationship between AHR expression and the regulation of the tricarboxylic acid cycle in mitochondria.

### AHR is positively associated with several genes involved in the mitochondrial electron transport chain

**Figure 3:**
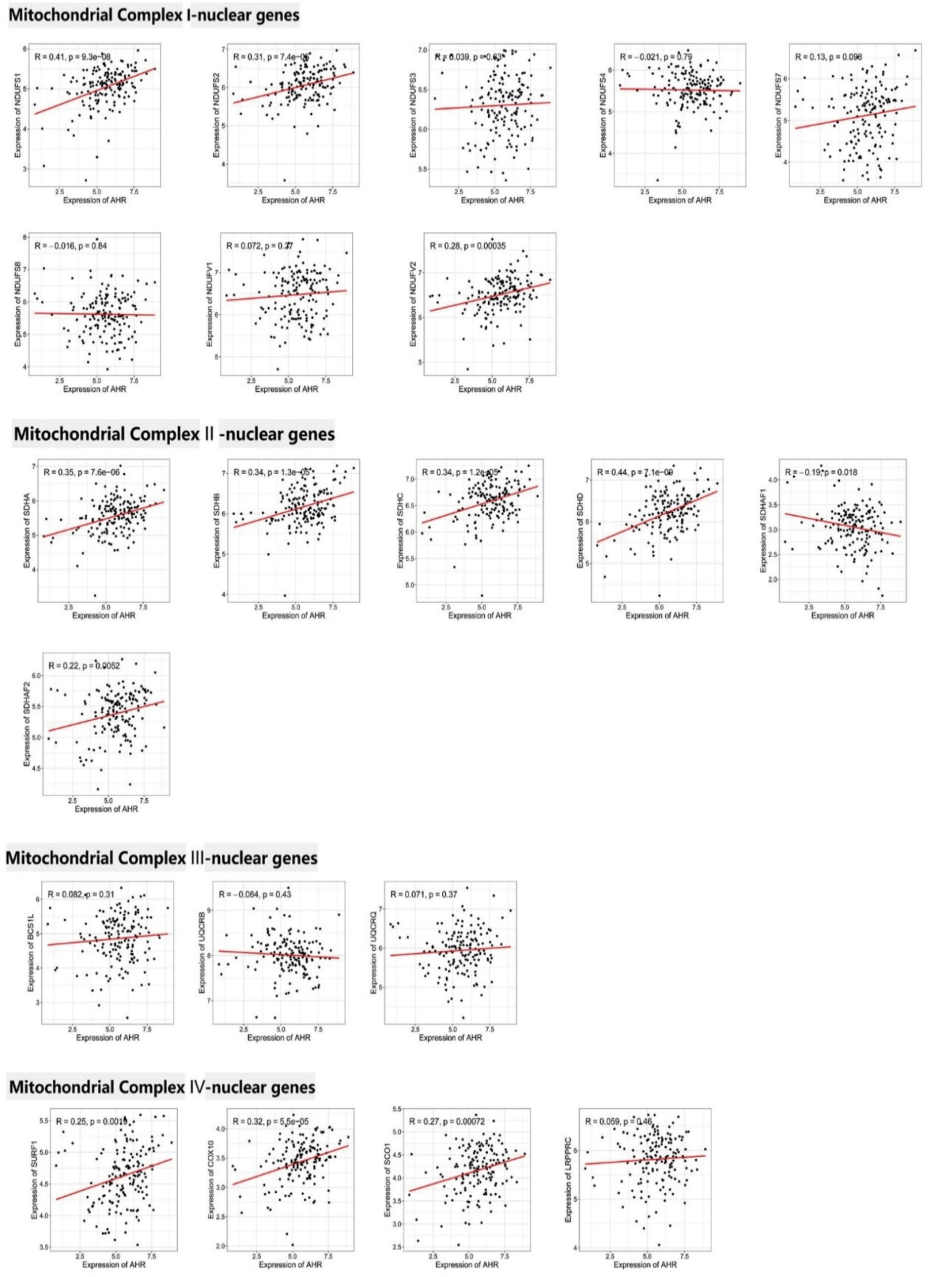
Figure 3 AHR is positively correlated with several genes involved in the mitochondrial electron transport chain The mitochondrial electron transport chain plays important roles in oxidative phosphorylation and is made up of four main compounds. The data indicated that AHR expression is positively associated with many mitochondrial electron transport chain genes.

Mitochondrial electron transport chain (ETC) play key roles in the production of ATP during oxidative phosphorylation and are made up of four compounds, compound I (NADH dehydrogenase compound), compound II (succinate dehydrogenase compound), compound III (cytochrome reductase compound), and compound IV (cytochrome c oxidase compound). In this study, we detected the correlation between AHR expression and *ETC* genes. The data indicated that AHR expression is positively associated with some mitochondrial electron transport chain genes (NDUFS1 R=0.41; NDUFS2 R=0.31; NDUFV2 R=0.28; SDHA R=0.35; SDHB R=0.34; SDHC R=0.34; SDHD R=0.44; SDHAF1 R=0.19; SDHAF2 R=0.22; SURF1 R=0.25; COX10 R=0.32; SCO1 R=0.27) ( Figure 3) **.**

### Activation of AHR promotes mitochondrial oxidative phosphorylation, while inhibition of AHR reduces mitochondrial oxidative phosphorylation

**Figure 4:**
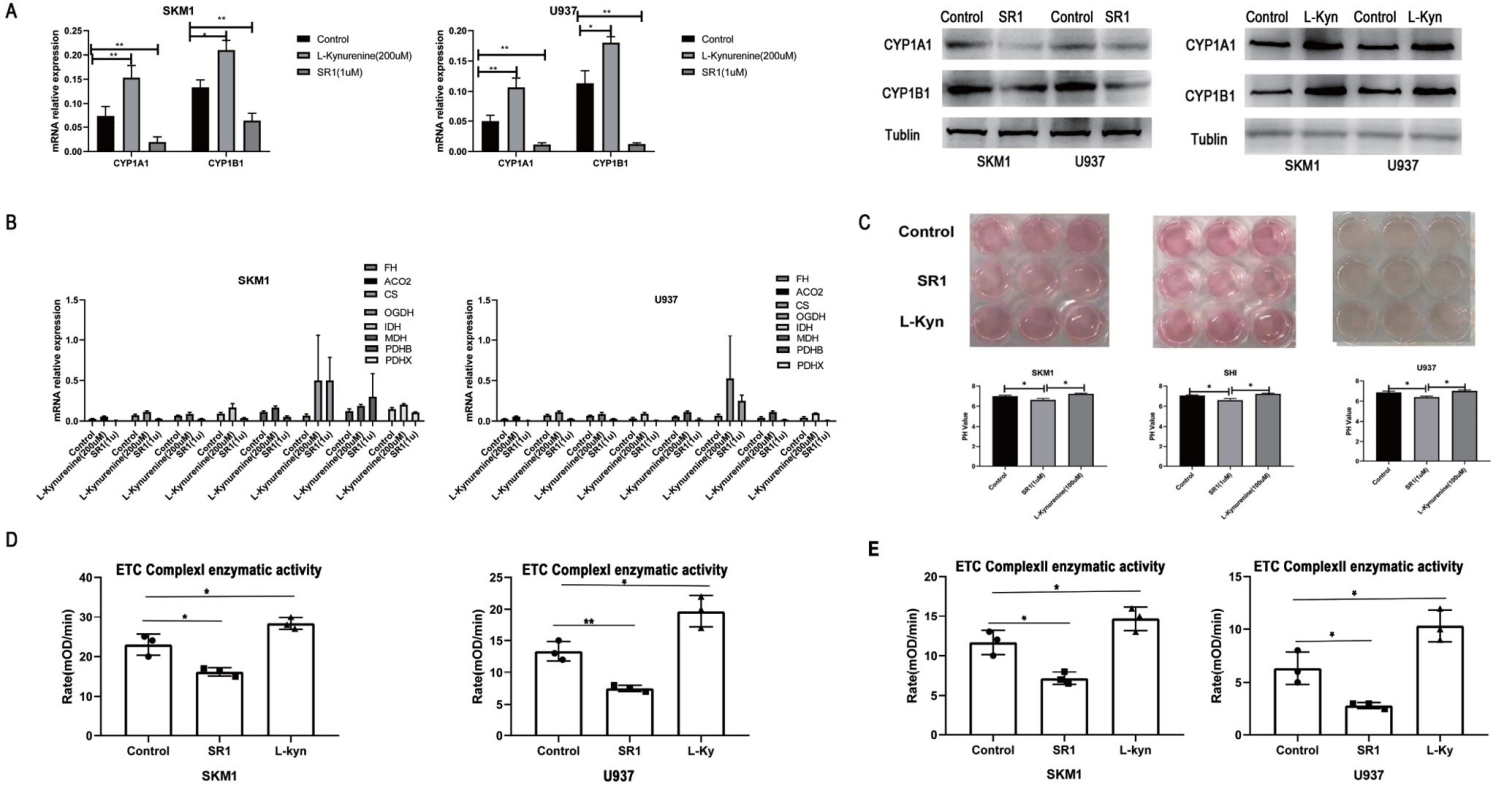
Figure 4 AHR activation mediates oxidative phosphorylation in AML cells (A) SKM1 and U937 cells were cultured with SR1 (1 μM) or L-Kyn (200 μM). The downstream AHR molecules, CYP1A1 and CYP1B1, were assessed for mRNA and protein expressions at 24 h or 48 h using qPCR and western blot analysis, respectively. The expression levels of CYP1A1 and CYP1B1 were found to be reduced upon AHR inhibition with SR1 (1 μM) in comparison to the control group, while they increased in the L-Kyn (200 μM) groups. (B) In AML cells cultured with SR1 (1 μM) for 24 h, the mRNA expressions level of mitochondrial oxidative phosphorylation molecules were lower than those in the control group. Conversely, activation of AHR with L-Kyn (200 μM) led to an increase in the expression levels of these mitochondrial oxidative phosphorylation molecules. (C) After 72 h, the cell culture medium treated with SR1 exhibited a significantly lighter color compared to that in the L-Kyn group or the normal control group, accompanied by a decrease in pH. (D,E) The results demonstrated that activating AHR with L-Kyn (200 μM) enhances the enzymatic activity of ETC Complexes I/II, while inhibiting AHR with SR1 reduces the enzymatic activity of ETC Complexes I/II. *P<0.05, **P<0.01.

In the study, we used SR1 (1 μM) to inhibit the AHR signaling pathway, while L-Kyn (200 μM) was used to activate AHR. After 48 h of incubation, we assessed the activation of the AHR signaling pathway by measuring the expression levels of CYP1A1 and CYP1B1 molecules using qPCR and western blot analysis. The results showed that the expressions of CYP1A1 and CYP1B1 were significantly increased in the L-Kynurenine group compared to those in the control group, whereas their expressions were decreased in the SR1 group ( Figure 4A). We also examined the effect of AHR activation and inhibition on the expression of mitochondrial oxidative phosphorylation genes ( *FH*, *ACO2*, *CS*, *OGDH*, *IDH*, *MDH*, *PDHB*, and *PDHX*) at 24 h. The data demonstrated that activating AHR led to an elevation in the expressions of these genes, while inhibiting AHR resulted in decreased expressions of these genes ( Figure 4B). Moreover, we observed that the color of the culture medium (containing phenol red) changed after AHR activation or inhibition. After 72 h, the culture medium in the SR1 group appeared significantly lighter in color compared to that in the L-Kynurenine group or normal control group, accompanied by a decrease in pH value ( Figure 4C). We hypothesized that blocking AHR signals may alter cellular mitochondrial metabolism, leading to a reduction in oxidative phosphorylation and an increase in acidity. Additionally, we found that activating AHR with L-Kyn (200 μM) for 48 h enhanced the enzymatic activity of ETC Complexes I and II, while inhibiting AHR with SR1 (1 μM) had the opposite effect ( Figure 4D,E).

### Decreased AHR expression results in diminished mitochondrial oxidative phosphorylation

**Figure 5:**
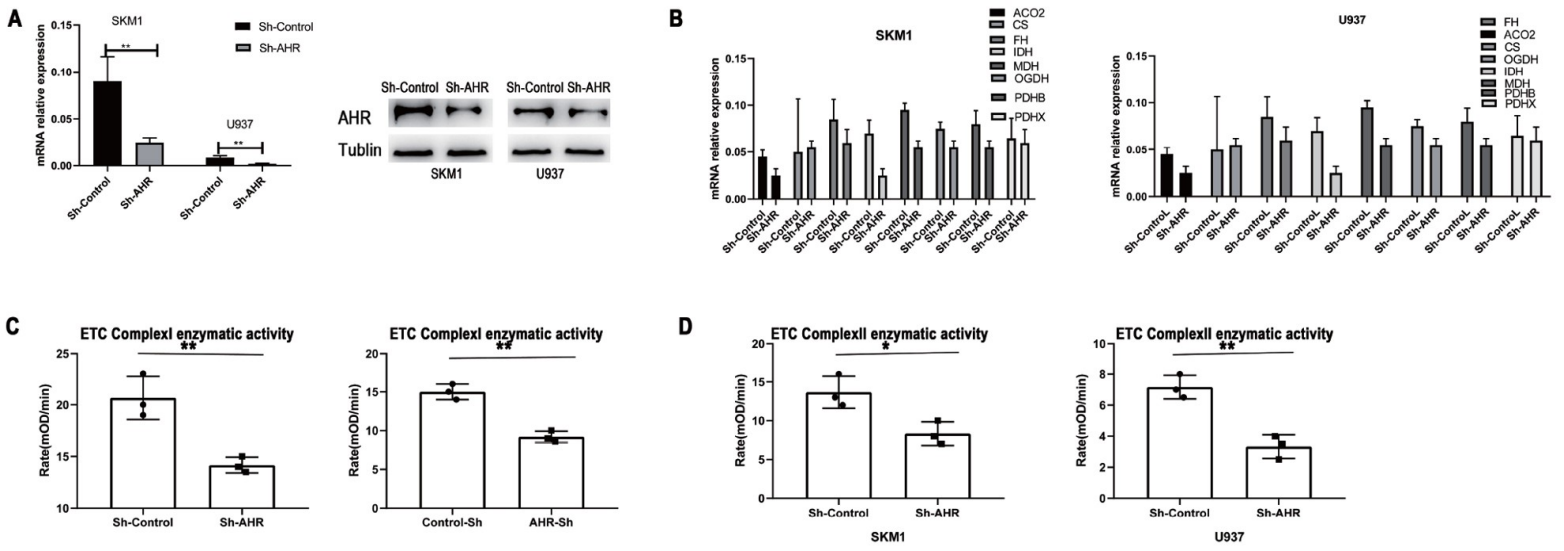
Figure 5 Reducing AHR expression in AML cells decreases mitochondrial oxidative phosphorylation (A) After reducing AHR expression with Sh-AHR, the mRNA/protein of AHR expression is significantly decreased compared to that in the control group. (B) Decreased AHR expression reduced mitochondrial oxidative phosphorylation gene expression compared with the control group. (C,D) Reduced AHR expression abated ETC Complexes I/II enzymatic activity significantly. *P<0.05, **P<0.01.

shAHR was utilized to attenuate the expression of AHR. AHR expression was assessed at 24 h using RT-PCR and at 48 h using western blot analysis ( Figure 5A). Following the knockdown of *AHR*, the expressions of mitochondrial oxidative phosphorylation genes were decreased compared to those in the control group ( Figure 5B). To further confirm the involvement of AHR in mitochondrial oxidative phosphorylation, we investigated the enzymatic activity of ETC Complexes I and II after reducing AHR expression. The results indicated that reduced expression of AHR significantly diminished the enzymatic activity of ETC Complexes I and II ( Figure 5C,D).

### Increased expression of AHR leads to an increase in mitochondrial oxidative phosphorylation

**Figure 6:**
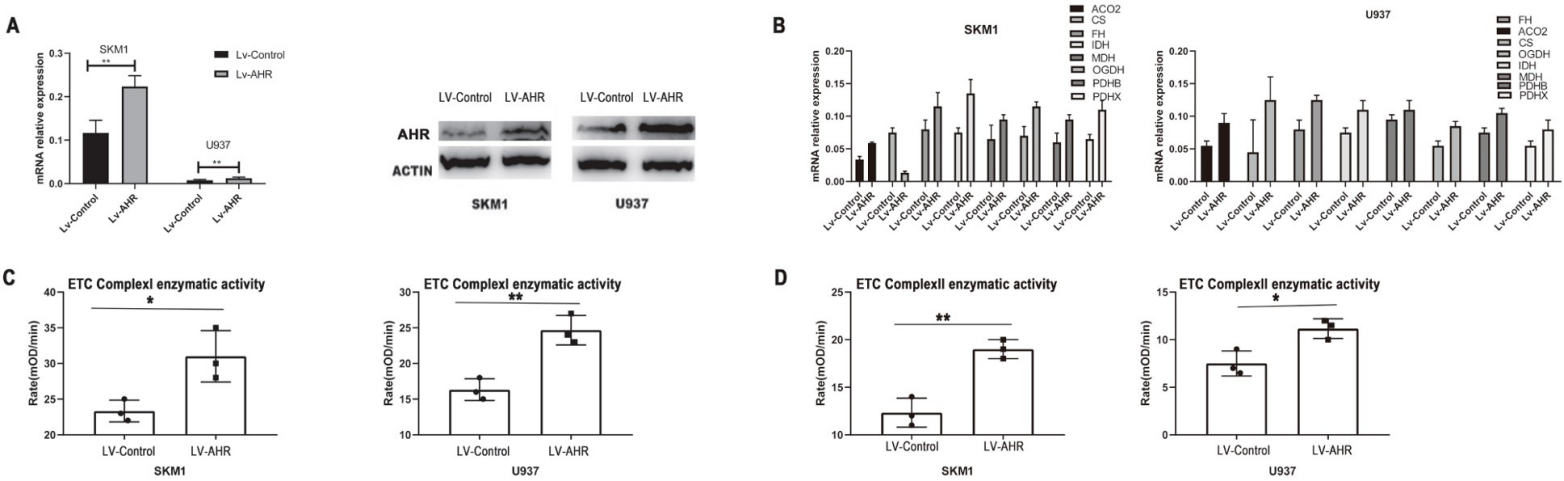
Figure 6 Increased AHR expression in AML cells enhances mitochondial oxidative phosphorylation (A) AHR expression significantly increased at both the mRNA and protein levels following transfection of AML cells with a retrovirus carrying the AHR gene. (B) Increasing AHR expression resulted in an upregulation of mitochondrial oxidative phosphorylation gene expression. (C,D) ETC Complexes I/II enzymatic activity showed a significant increase after AHR expression was elevated. *P<0.05, **P<0.01.

In order to further investigate the role of AHR in AML, we conducted an experiment where AML cells were transfected with a retrovirus carrying the *AHR* gene. As a result, the expression of AHR was significantly increased at both the mRNA and protein levels ( Figure 6A). The enhanced expression of AHR led to an increase in the expressions of mitochondrial oxidative phosphorylation genes ( Figure 6B). Moreover, the enzymatic activity of ETC Complexes I and II showed a significant increase after the elevation of AHR expression ( Figure 6C,D). These findings indicated that increasing the expression of AHR enhances mitochondrial oxidative phosphorylation in AML cells.

### AHR mediates cytarabine resistance

**Figure 7:**
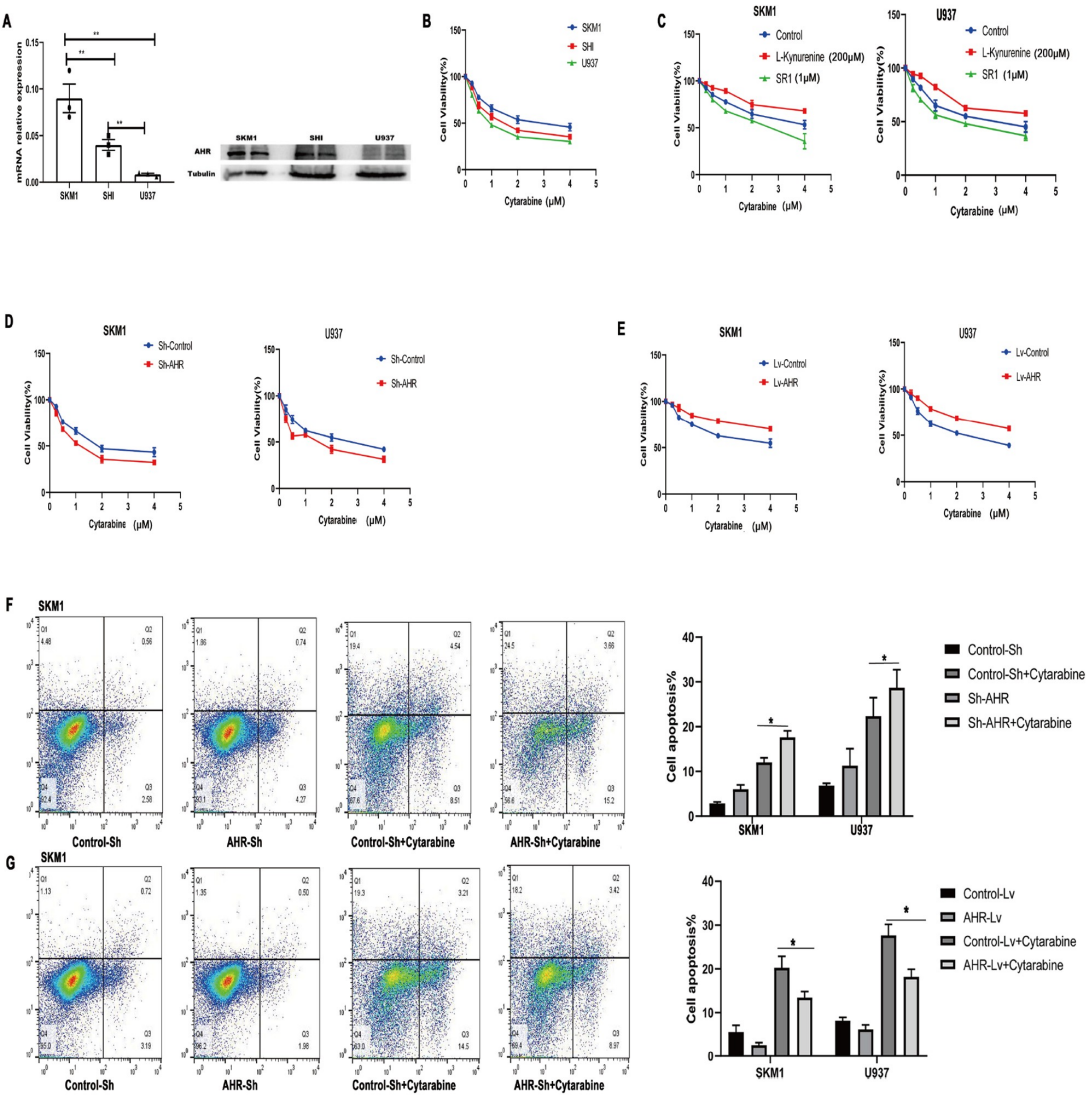
Figure 7 AHR is involved in cytarabine resistance (A,B) As for AML cells, higher expression of AHR was correlated with increased resistance to cytarabine. (C) Activating AHR with L-Kyn (200 μM) enhances the resistance of AML cells to cytarabine, while inhibition of AHR with SR1 (1 μM) increases the sensitivity of AML cells to cytarabine. (D) Knockdown of AHR results in increased sensitivity of AML cells to cytarabine. (E) Conversely, enhancing AHR expression attenuates the sensitivity of AML cells to cytarabine. (F,G) Reduced AHR expression enhances apoptosis induced by cytarabine, while increased AHR expression has the opposite effect. *P<0.05, **P<0.01.

In the study, we utilized RT-PCR and western blot analysis to detect the expression of AHR in various AML cell lines. We found that higher AHR expression corresponded to increased resistance to cytarabine ( Figure 7A,B). We further activated AHR with L-Kyn (200 μM) and observed enhanced resistance of AML cells to cytarabine. Conversely, inhibiting AHR with SR1 (1 μM) increased the sensitivity of AML cells to cytarabine ( Figure 7C). Knockdown of *AHR* resulted in increased sensitivity to cytarabine ( Figure 7D), while enhancing *AHR* expression attenuated the sensitivity of AML cells to cytarabine ( Figure 7E). We also examined the impact of AHR expression on cytarabine-induced apoptosis, and found that reducing AHR expression enhanced cytarabine-induced apoptosis ( Figure 7F), whereas increased AHR expression had the opposite effect ( Figure 7G). These findings suggested that AHR plays a crucial role in mediating cytarabine resistance in AML cells.

## Discussion

AML is a hematological malignancy characterized by a loss of differentiation and rapid proliferation of malignant cells [33]. Despite advancements in chemotherapy and the introduction of new drugs, the prognosis for AML patients continues to be unfavorable [34].

AHR is a member of the classical helix-loop-helix transcription factor family [2]. It has been established that activating AHR plays a critical role in cancer cell development, invasion, and metastasis [ 4‒ 6]. Endogenous ligands of AHR include tryptophan metabolites, with the metabolism of tryptophan being mediated through two rate-limiting enzymes: TDO and IDO [ 7, 35]. Recent studies suggested that interleukin-4-induced-1 (IL4I1) is more frequently associated with AHR activity than IDO1 or TDO2 [10]. Activating AHR enhances the expressions of cytochromes P450 (CYP1A1, 1A2, and 1B1), and increased levels of CYP1A1 and CYP1B1 indicate AHR activation [ 7, 13]. The role of AHR in AML is controversial, with some studies suggesting that AHR activation promotes immune evasion by AML cells, while others suggest that it inhibits leukemic stem and progenitor cell (LSPC) proliferation in AML [ 36, 37]. In our study, we found that the molecules of the AHR signaling pathway (IDO1/IL4i1/AHR/CYP1B1) are elevated in AML patients. Furthermore, increased expression of IL4i1 was found to be indicative of an unfavorable prognosis in AML patients. We also detected the expression of AHR in AML patients and found that the expression level of AHR in BMMCs was significantly increased in AML patients, which was linked to a worse overall survival rate. These results suggested that molecules in AHR signaling pathway express aberrantly in AML, which may promote the progression of AML.

Cancer cells are known to have increased glycolysis compared to normal cells, which has led to the assumption that OXPHOS is reduced in tumors [38]. However, recent evidence suggests that OXPHOS plays a critical role in delivering the bioenergetic and macromolecular anabolic requirements of cancer cells [ 19, 39]. Francois *et al*. [20] found that mitochondrial oxidative phosphorylation plays a key role in the maintenance of tumor cell stemness. There is also mounting evidence indicating that AHR plays important roles in mitochondrial oxidative phosphorylation [ 27, 28]. AHR signaling is involved in maintaining cellular homeostasis by modulating mitochondrial activity and oxidative stress [ 30‒ 32]. In our study, we found that AHR expression is associated with mitochondrial OXPHOS molecules. We observed that mitochondrial oxidative phosphorylation was elevated after activating AHR with L-Kyn (200 μM) and decreased after inhibiting AHR with SR1 (1 μM). To further confirm the role of AHR in mitochondrial oxidative phosphorylation, we reduced and enhanced AHR expression. The results demonstrated that decreased AHR expression in AML cells reduced mitochondrial oxidative phosphorylation, while increased AHR expression in AML cells enhanced mitochondrial oxidative phosphorylation. These findings suggested that AHR mediates oxidative phosphorylation and is involved in the development of AML.

Despite improvements in chemotherapy, the full remission rate for AML patients is still low, and drug resistance remains a significant challenge in AML treatment [40]. The standard treatment for AML consists of intensive induction and consolidation chemotherapy, with cytarabine being the main drug used in treatment [ 41, 42]. However, the 5-year survival rate for AML patients is less than 30%, and cytarabine resistance is one of the reasons for this [43]. It has been shown that AraC-resistant AML cells exhibit high levels of OXPHOS [43]. Furthermore, inhibiting OXPHOS in AML cells strongly enhances the anti-leukemic effects of AraC [ 41, 44].

In summary, we found that AHR was aberrantly expressed in AML patients and played an important role in mitochondrial oxidative phosphorylation, which might be associated with cytarabine resistance. These findings may contribute to a deeper understanding of the involvement of the AHR signaling pathway in AML.
